# Aerosol generating procedures (AGP) and risk of transmission of acute respiratory diseases (ARD): a systematic review

**DOI:** 10.1186/1753-6561-5-S6-P91

**Published:** 2011-06-29

**Authors:** K Tran, K Cimon, M Severn, C Pessoa-Silva, J Conly

**Affiliations:** 1 CADTH, Ottawa, Canada; 2 WHO, Geneva, Switzerland

## Introduction / objectives

The risk of transmission of ARDs to HCWs from AGPs is not fully known. We sought to determine the evidence for the risk of transmission of acute ARDs to HCWs caring for patients undergoing and not undergoing AGPs.

## Methods

We searched PubMed, Medline, Embase, Cinahl, the Cochrane Library, Univ of York CRD databases, EuroScan, LILACS, Indian Medlars, Index Medicus for SE Asia,health technology agencies and the Internet in all languages for articles from 01/01/1990 – 22/10/2010. Abstracts and full text articles were screened and included using pre-defined criteria. Disagreements were resolved by consensus and a 3^rd^ reviewer. Data were extracted and verified by a 2^nd^ reviewer. The outcome of interest was risk of ARD transmission. The quality of evidence was rated using the GRADE system.

## Results

We identified 5 case-control and 5 retrospective cohort studies which evaluated transmission of SARS to HCWs. Procedures with an ­increased risk of transmission included [n; pooled OR(95%CI)] tracheal intubation [n=8; 6.2 (3.4, 11.3)], non-invasive ventilation [n=2;OR 3.1(1.4, 6.8)], tracheotomy [n=1; 4.2 (1.5, 11.5)] and manual ventilation before intubation [n=1;OR 2.8(1.3, 6.4)]. Other intubation procedures, ET aspiration, suction of body fluids, bronchoscopy, nebulizer treatment, administration of O^2^, high flow O^2^, manipulation of O^2^ mask or BiPAP mask, defibrillation, chest compressions, insertion of NG tube, and collection of sputum were not significant.

## Conclusion

Our findings suggest that some procedures have been associated with increased risk of SARS transmission to HCWs with the most consistent association across multiple studies identified with tracheal intubation. These findings must be interpreted in the context of the very low quality of the studies.

## Disclosure of interest

K. Tran : None declared, K. Cimon: None declared, M. Severn : None declared, C. Pessoa-Silva: None declared, J. Conly Other Clincal expert for other CADTH projects and speaker honoraria from Industry related to new antimicrobials.

